# Management of psoriasis in women 18 to 45 years of age in Australia and Japan: insights from patient and dermatologist surveys

**DOI:** 10.1097/JW9.0000000000000189

**Published:** 2025-01-02

**Authors:** Yukie Yamaguchi, Lynda Spelman, Yoko Mizutani, Bartosz Lukowski, Alfred Lanzafame, Annika Smith

**Affiliations:** a Department of Environmental Immuno-Dermatology, Yokohama City University Graduate School of Medicine, Yokohama, Japan; b Veracity Clinical Research and Probity Medical, Queensland, Australia; c Department of Dermatology, Gifu University Graduate School of Medicine, Gifu, Japan; d UCB, Tokyo, Japan; e UCB, Victoria, Australia; f St Vincent’s Hospital, Westmead Hospital, University of Sydney, New South Wales, Australia

**Keywords:** family planning, pregnancy, psoriasis, survey, tumor necrosis factor inhibitors

## Abstract

**Background:**

A psoriasis (PSO) diagnosis may pose specific treatment challenges for women of childbearing age (WoCBA) who are considering pregnancy, are pregnant, or have just given birth.

**Objective:**

To report perspectives of WoCBA with PSO regarding pregnancy and dermatologists about the disease management of these women in Australia and Japan.

**Methods:**

Online surveys were completed by women aged 18 to 45 years who were pregnant within the past 5 years with moderate to severe PSO and dermatologists.

**Results:**

In Japan (*n* = 31) and Australia (*n* = 27), most WoCBA with PSO did not feel adequately informed about pregnancy planning and had concerns regarding the safety of tumor necrosis factor inhibitors (TNFi) when used during pregnancy. Dermatologists (Australia: *n* = 40; Japan: *n* = 97) also had safety concerns around prescribing TNFi during pregnancy, and most were impartial toward or not at all comfortable with prescribing TNFi to women who were pregnant or actively planning pregnancy. Dermatologists felt that more safety data on pregnancy, lactation, and pediatric outcomes 5 years postdelivery would increase their comfort with prescribing TNFi.

**Limitations:**

Limitations included small respondent size, a lack of formal validation for questionnaires, recall bias among participants, and generalizability of results to all WoCBA with PSO. Response rates of survey participants were also not collected.

**Conclusion:**

Additional safety information can help address concerns about biologic use (including TNFi) in WoCBA, enabling dermatologists to make informed treatment recommendations in such patients.

**What is known about this subject in regard to women and their families?**

- As diagnosis and initiation of psoriasis (PSO) treatment often overlap with peak reproductive years for women aged 18 to 45 years and there are limited treatment options that are compatible with pregnancy or breastfeeding, PSO may pose specific treatment challenges for women of childbearing age (WoCBA) who are planning to become pregnant, are pregnant, or have just given birth.
- It is therefore important to recognize patient and dermatologist-perceived barriers to treatment among WoCBA with PSO to guide disease management, particularly regarding tumor necrosis factor inhibitors (TNFi) as these are one of the most commonly used classes of biologics for moderate to severe PSO. Furthermore, the knowledge of safety data on TNFi in this population is limited in Australia and Japan.

**What is new from this article as messages for women and their families?**

- Concerns and gaps in information regarding family planning were common among WoCBA with PSO, and dermatologists had safety concerns around prescribing TNFi during pregnancy.
- There is a need to address dermatologists’ underlying concerns regarding disease management among WoCBA with PSO; as dermatologists’ perceptions can ultimately influence the medical care patients receive, raising awareness among dermatologists can aid in elevating the standard of care for WoCBA with PSO.
- Educational resources that improve dermatologists’ access to up-to-date, accurate TNFi safety data can help dermatologists support their patients in making informed decisions balancing disease management without compromise to family planning, and to discuss strategies that would proactively safeguard the health of both mother and child.

## Introduction

Psoriasis (PSO) is a chronic inflammatory dermatological condition that can have a significant negative impact on patients’ quality of life and confer a high financial burden.^1^

Diagnosis and initiation of treatment often overlap with peak reproductive years for women between the ages of 18 to 45 years (women of childbearing age [WoCBA])^2,3^ and may therefore pose specific treatment challenges for WoCBA who are planning to become pregnant, are pregnant, or have just given birth.^2^ Notably, women with more severe forms of PSO, such as generalized pustular PSO during pregnancy, may be particularly adversely affected as this variant of the disease can be life-threatening for both mother and fetus.^4^ Furthermore, PSO as a comorbidity during pregnancy has been associated with adverse pregnancy outcomes and disease flares postpartum, which can be exacerbated by untreated or uncontrolled disease.^2,5–7^

A recent survey of WoCBA with PSO in Europe observed that family planning and childbearing aspirations were affected by their disease.^8^ Although the advent of improved therapies for inflammatory diseases has enabled many female patients to consider starting a family, not all treatment options are compatible with pregnancy or breastfeeding.^2,9^ As such, navigating treatment for WoCBA can be complex.^2^

Tumor necrosis factor inhibitors (TNFi) are one such treatment option for PSO.^10–13^ As TNFi are alternatives where topical agents, phototherapy, and other systemic therapies (eg, methotrexate and cyclosporin) have been insufficient or ineffective,^10,11^ TNFi remain a valuable option in the PSO treatment landscape. In contrast with biologics such as interleukin (IL)-17 or IL-23 inhibitors, some TNFi have specifically demonstrated suitability for consideration during pregnancy and breastfeeding in the treatment of PSO.^14–16^ Despite this, rates of TNFi use among WoCBA with PSO remain particularly low,^2,17^ and data on perspectives of WoCBA and dermatologists from Australia and Japan are limited. Hence, to optimize disease management, there is a need to recognize patient and dermatologist-perceived barriers to treatment in WoCBA with PSO.

This online survey therefore sought to report the attitudes and perceptions of WoCBA (18–45 years) with PSO regarding pregnancy and that of dermatologists regarding the management of PSO among these women in Australia and Japan.

## Methods

### Survey design

Patients were recruited from consumer panels by Hummingbird Insight (Sydney, Australia) to participate in an online survey conducted from September to October 2018. Dermatologists were contacted and invited by IQVIA (Sydney, Australia, and Tokyo, Japan, for the respective countries) to complete an online survey conducted from October to December 2020. The full survey design and questionnaires are described in Supplemental Material, http://links.lww.com/IJWD/A61.

### Participants

#### Patients

Eligible participants included women aged 18 to 45 years diagnosed with PSO (any type, including plaque PSO) in Australia and Japan, with self-reported moderate to severe disease. Additionally, eligible patients were not pregnant at the time of enrollment but had been pregnant within the past 5 years and had used medication (including TNFi) in their disease history.

#### Dermatologists

Participants included dermatologists from Australia and Japan who were managing PSO in at least one female patient aged 18 to 45 years using biologic agents, including TNFi, at the time of the survey. In addition, eligible dermatologists from Japan had to have practices at an accredited clinical facility approved for biologics use by the Japanese Dermatological Association. Of these, only hospital-based dermatologists were included.

### Statistical analysis

Data from both quantitative surveys were summarized descriptively for Australia and Japan and reported as proportions of patients or dermatologists.

### Compliance with ethics guidelines

The market research companies that conducted the study acted in accordance with the appropriate codes of conduct regarding anonymity and confidentiality and are also fully compliant with the Data Protection Act of the respective countries. Since this was a market research study, prior approval of the protocol by an ethics committee was not required. As the market research companies involved are based in Australia and Japan, the study was conducted in accordance with Australian and Japanese market research guidelines, including the obtaining of informed consent and adherence to ethical reporting standards.

## Results

### Patient survey

#### Participant demographics and disease characteristics

A total of 58 women (Australia: *n* = 27; Japan: *n* = 31) participated in the survey (see Supplemental Material, http://links.lww.com/IJWD/A61). Most patients were aged 31 to 40 years. In Australia, 59% (*n* = 16/27) of patients were diagnosed with PSO during pregnancy, versus 94% (*n* = 29/31) of patients from Japan who were diagnosed before pregnancy. Most patients also reported their disease to be of moderate severity (Australia: 67%, *n* = 18/27; Japan: 94%, *n* = 29/31). In Australia and Japan, 56% (*n* = 15/27) and 19% (*n* = 6/31) of patients used TNFi (for any indication) before pregnancy, respectively.

#### Overall information gaps

**Fig. 1.**
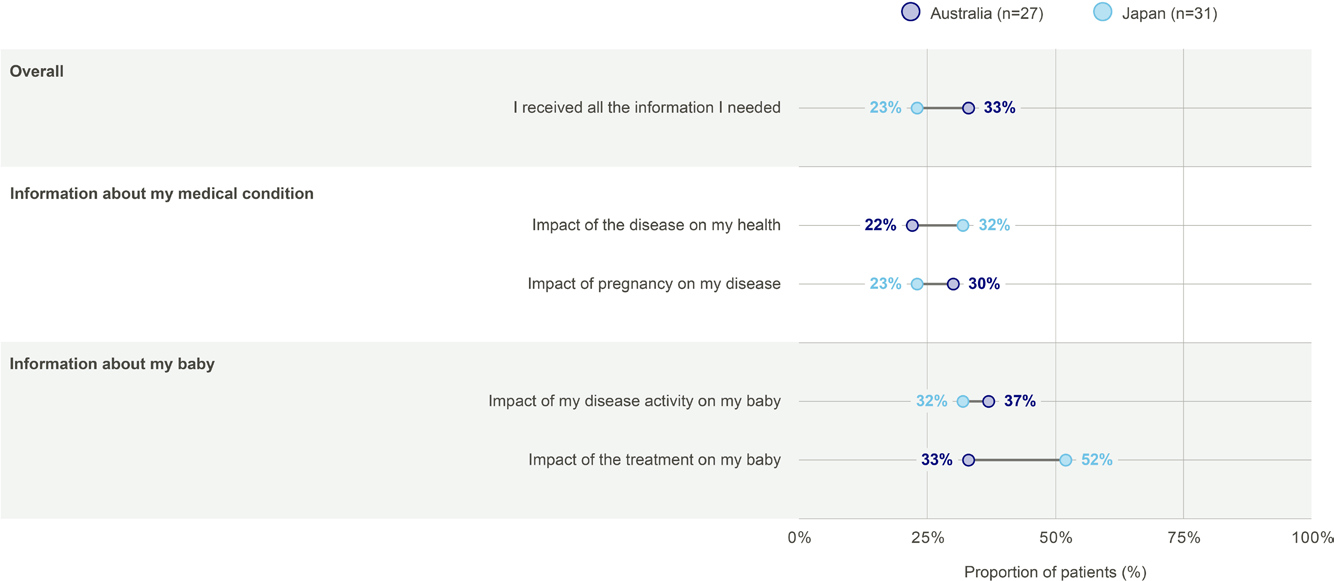
Sufficiency of information provided by HCPs. Data should be interpreted with caution due to small *n* numbers. “Prior to pregnancy, did you receive all the information you needed from your healthcare professional?” All possible answers are shown, except for the “Other” category that recorded no responses from Australian patients and 3 (10%) responses from Japanese patients. Multiple responses were possible. HCP, healthcare professionals.

Prior to pregnancy, 33% (*n* = 9/27) of patients from Australia and 23% (*n* = 7/31) of patients from Japan felt they received all the information they needed (Fig. 1). Similar proportions of patients from Australia and Japan felt they received sufficient information about their medical condition and the impact of disease activity on their babies. In Australia, 33% (*n* = 9/27) of patients felt they received sufficient information about the impact of their PSO treatment on their baby, versus 52% (*n* = 16/31) of patients from Japan.

#### Concerns prior to pregnancy

**Fig. 2.**
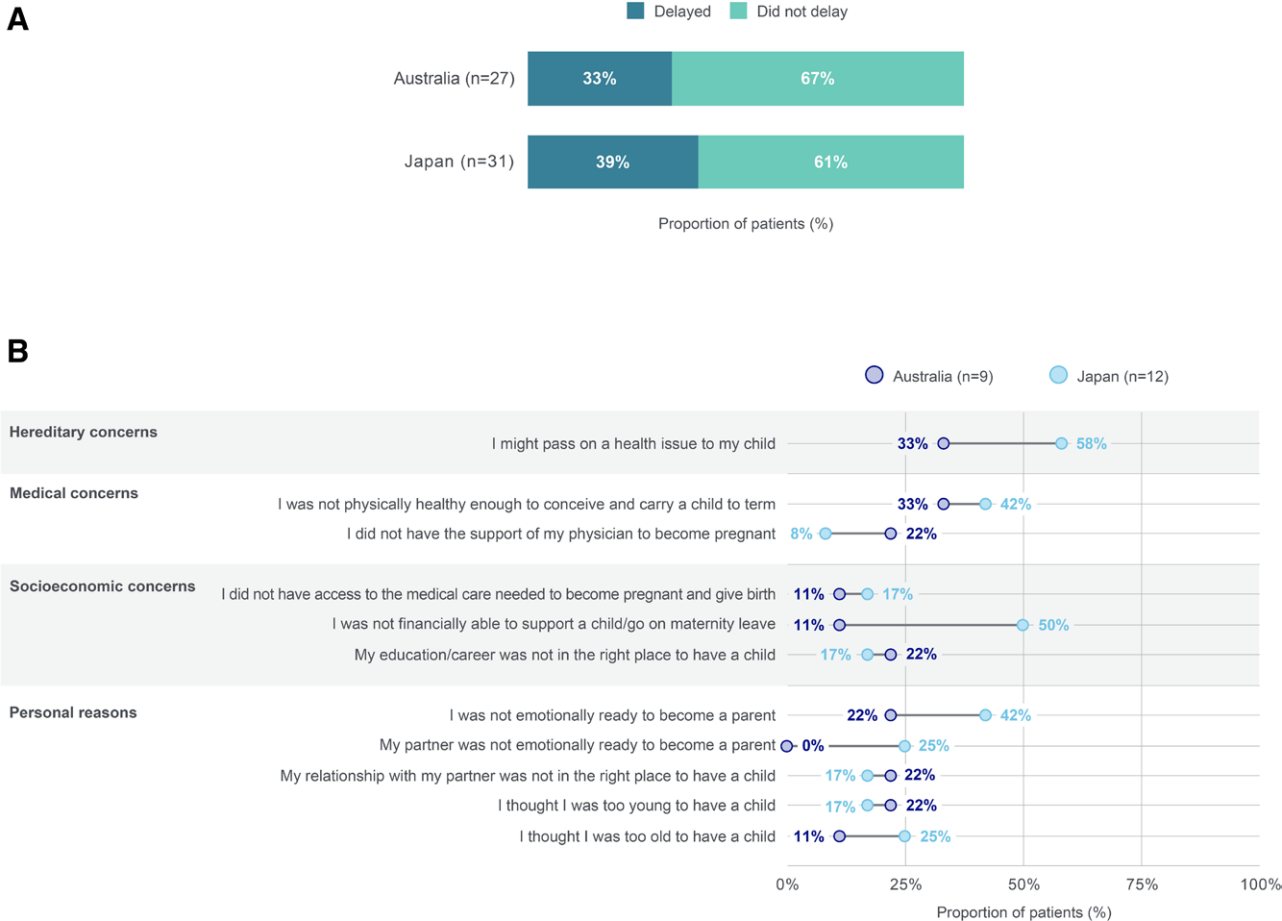
Family planning among women with PSO. (A) Proportion of women with PSO that delayed their decision to become a mother. (B) Concerns of women with PSO that delayed their decision to become a mother. Data should be interpreted with caution due to small *n* numbers. (A) “Did you have any concern that delayed your decision to become a mother for your most recent pregnancy?”; (B) “Which (if any) of the following concerns delayed your decision to become a mother for your most recent pregnancy?” Results reported for those who indicated “yes” in question (A). All possible answers are shown, except for the “None of the above” (1 [8%] response from a Japanese patient) and “Other” categories (no responses were recorded). Multiple responses were possible. PSO, psoriasis.

Approximately one-third of patients delayed their decision to have children for any reason (Fig. 2a). Specifically, among patients who delayed their decision to become a mother, hereditary concerns (passing on a health issue to their child) were the most common reason for doing so (Fig. 2b).

Regarding pregnancy planning (all data described are not shown in figures), this was most frequently first discussed at the time of treatment initiation for 37% (*n* = 10/27) patients from Australia and at the time of diagnosis of PSO for 35% (*n* = 11/31) patients from Japan. These discussions were more commonly initiated by the patient themselves or their partner (Australia: 52%, *n* = 14/27; Japan: 71%, *n* = 22/31), instead of being initiated by healthcare professionals (HCPs) (Australia: 33%, *n* = 9/27; Japan: 13%, *n* = 4/31). Approximately half of all patients did not have a treatment plan in place before trying to conceive (Australia: 56%, *n* = 15/27; Japan: 42%, *n* = 13/31).

#### Concerns during pregnancy

**Fig. 3.**
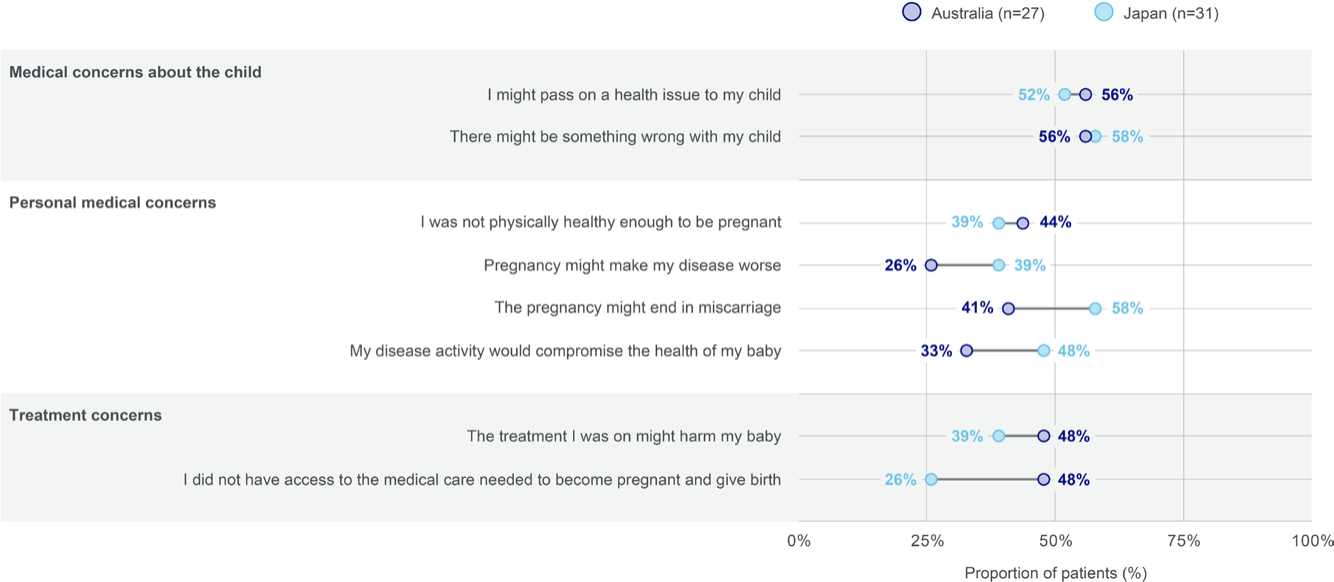
Concerns of women with PSO upon discovering they were pregnant. Data should be interpreted with caution due to small *n* numbers. “At the time you discovered you were pregnant, to what extent did you experience any of the following concerns?” Results were reported for those who indicated a score of 4 or 5 (out of 5) for their level of concern (1 representing “not at all concerned” and 5 representing “very concerned”). PSO, psoriasis.

Upon discovering they were pregnant, medical concerns about the child were the most common category of concern among patients (Fig. 3).

**Fig. 4.**
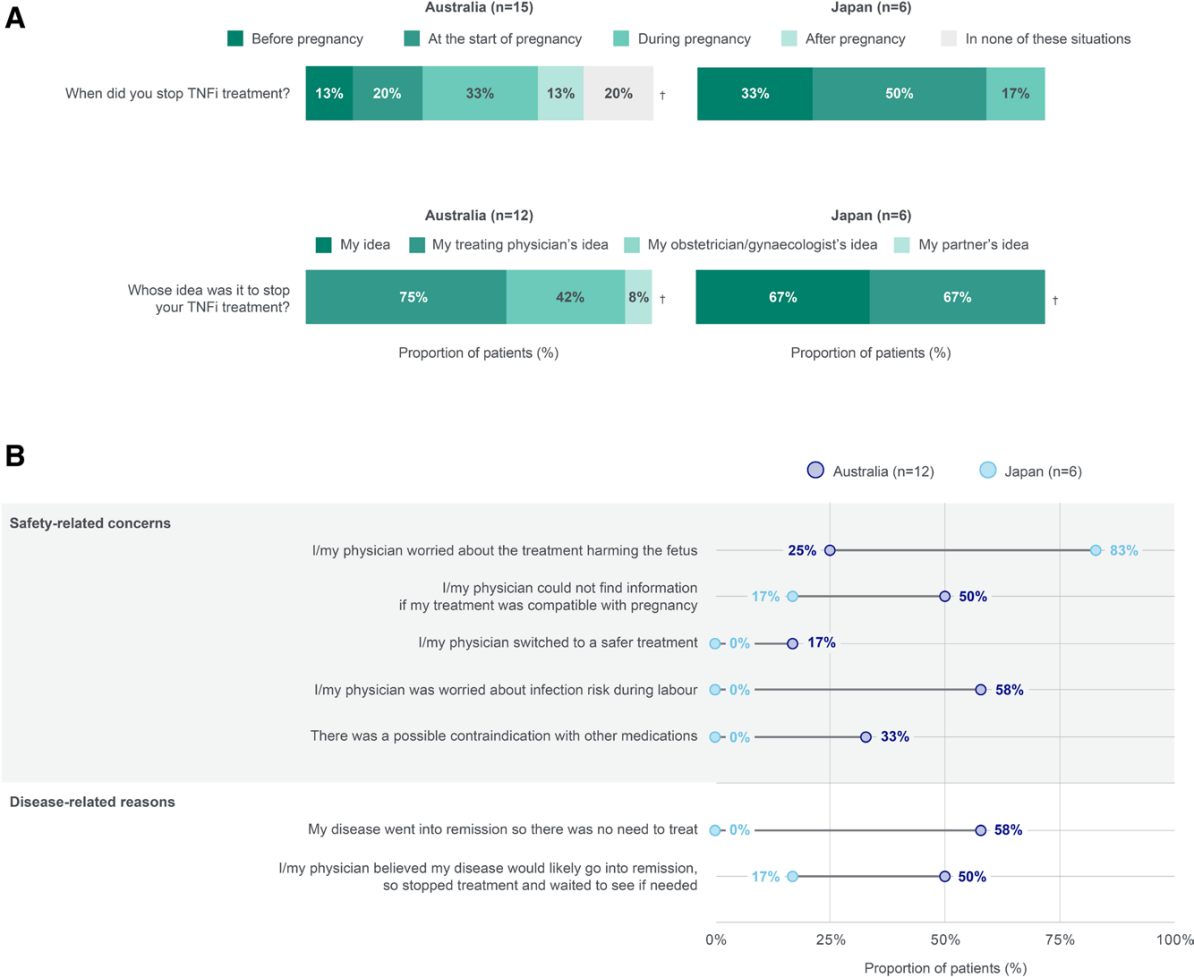
TNFi treatment for PSO during pregnancy. (A) When TNFi treatment was stopped and who initiated treatment cessation. (B) Patient-reported reasons for stopping TNFi treatment for PSO during pregnancy. Data should be interpreted with caution due to small *n* numbers. (A) “Did you stop TNFi treatment and if so when?,” ^†^These data do not add up to 100% as multiple answers were possible; “Whose idea was it to stop your TNFi treatment?”; (B) “Key reasons for stopping TNFi treatment” All possible answers are shown, except for the “Other” and “Physicians could not agree on the best plan” categories that recorded no answers. Multiple responses were possible. PSO, psoriasis; TNFi, tumor necrosis factor inhibitors.

Most patients discontinued TNFi treatment either at the start of pregnancy or during pregnancy (Fig. 4a), and treatment cessation was most commonly initiated by the treating physician. Safety-related concerns were among the most common reasons for stopping TNFi during pregnancy across patients (eg, infection risk during labor or harm to the fetus; Fig. 4b). Half of the patients and their physicians from Australia were also unable to find information on whether TNFi were compatible with pregnancy.

#### Concerns after giving birth

Of the patients from Australia and Japan, 85% (*n* = 23/27) and 45% (*n* = 14/31) had discussed the possibility of breastfeeding with their physician, respectively (data not shown). Furthermore, 33% (*n* = 7/21) and 50% (*n* = 15/30) of patients from Australia and Japan felt they had to make a choice between treatment and breastfeeding, respectively (data not shown).

Among patients from Australia and Japan, 30% (*n* = 8/27) and 90% (*n* = 28/31) breastfed their baby, respectively; the most common reasons for not breastfeeding were due to treatment-related concerns in Australia, and due to doctor/HCP recommendation not to breastfeed in Japan (see Supplemental Material, http://links.lww.com/IJWD/A61).

### Dermatologist survey

#### Participant demographics

A total of 137 dermatologists (Australia: *n* = 40; Japan: *n* = 97) participated in the survey (see Supplemental Material, http://links.lww.com/IJWD/A61). Most Australian dermatologists had practices located in a metropolitan area (90%, *n* = 35/39; information was not available for one respondent; data not shown). All Japanese dermatologists were hospital-based, of which 48% (*n* = 47/97) worked in a university hospital setting (data not shown).

#### Overall information gaps

**Fig. 5.**
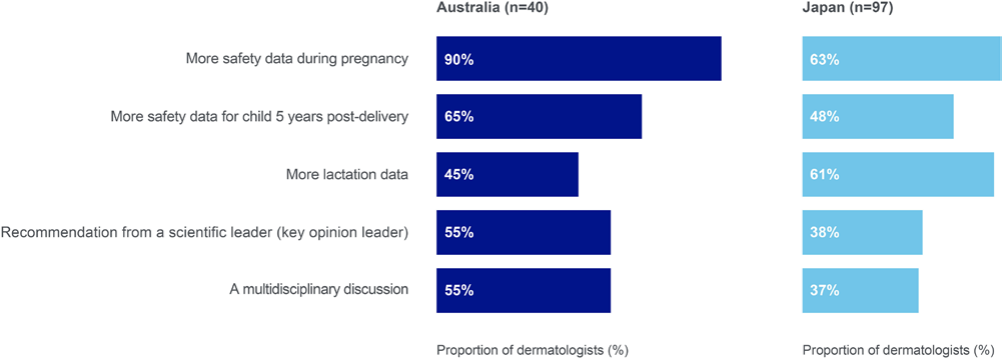
Factors increasing dermatologists’ comfort with prescribing TNFi in pregnant patients with PSO. “Hypothetically, what, if anything, would make you more comfortable with using TNFi agents among female patients between the age of 18–45 who may become pregnant in the future?.” Multiple responses were possible. PSO, psoriasis; TNFi, tumor necrosis factor inhibitors.

A need for more TNFi safety data during pregnancy, breastfeeding, and on pediatric outcomes 5 years postdelivery were reported as factors that would increase dermatologists’ comfort in using TNFi among female patients aged 18 to 45 years who may become pregnant in the future (Fig. 5).

#### Treatment goals and disease control

**Fig. 6.**
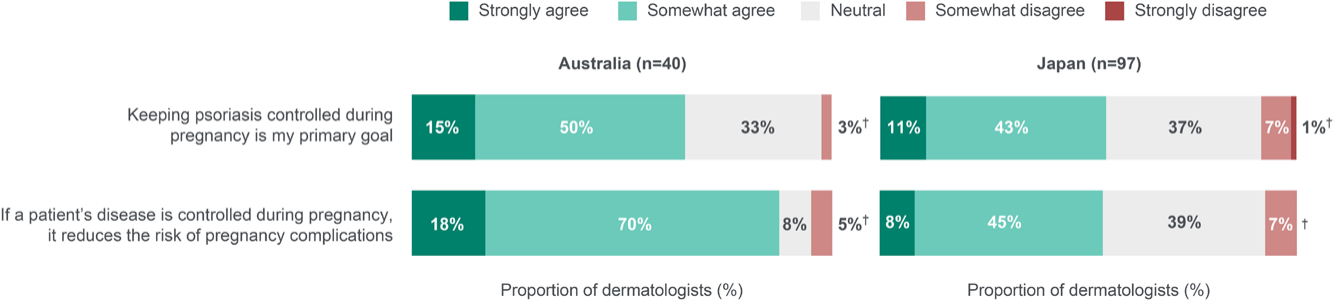
Dermatologists’ attitudes regarding PSO control and risk of complications in pregnant patients prescribed TNFi. ^†^These data do not add up to 100% due to a rounding error. PSO, psoriasis; TNFi, tumor necrosis factor inhibitors.

Over half of the dermatologists agreed that keeping patients’ PSO controlled during pregnancy was their primary goal and that the risk of pregnancy complications is reduced if PSO is controlled during pregnancy (Fig. 6).

#### Use of TNFi

**Fig. 7.**
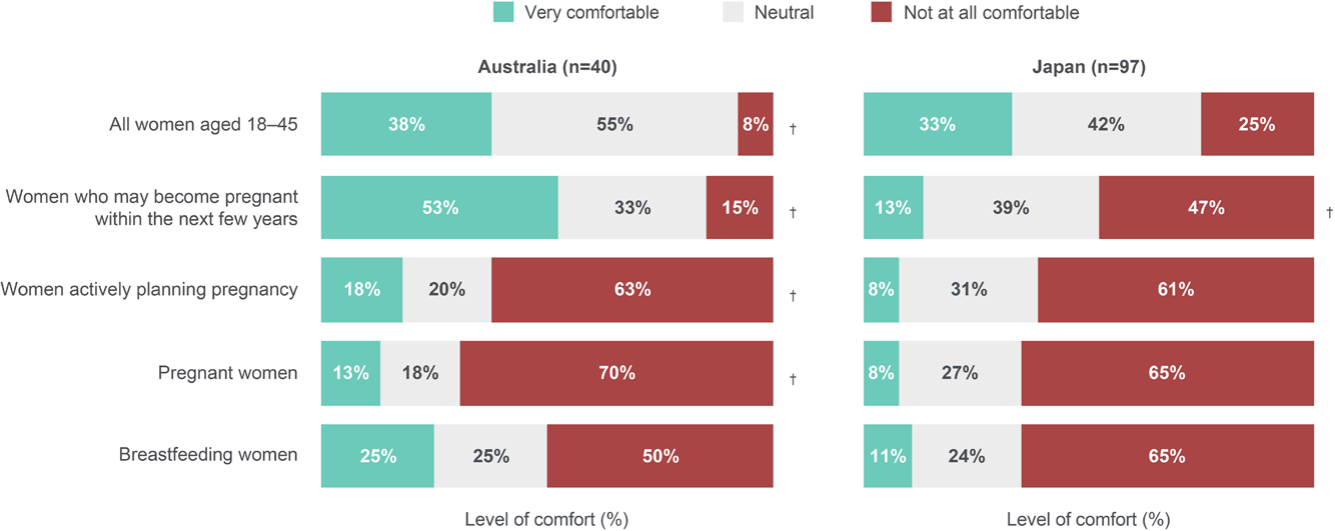
Dermatologists’ level of comfort with TNFi treatment in women aged 18–45 years with PSO. “How comfortable are you in prescribing TNFi therapy for the following types of PSO patients?.” Dermatologists from Japan were asked to disregard patients with psoriatic arthritis when responding. ^†^These data do not add up to 100% due to a rounding error. PSO, psoriasis; TNFi, tumor necrosis factor inhibitors.

Overall, 38% (*n* = 15/40) of dermatologists from Australia and 33% (*n* = 32/97) of dermatologists from Japan were “very comfortable” with prescribing TNFi to women aged 18 to 45 years (Fig. 7). Among dermatologists from Australia and Japan, 53% (*n* = 21/40) and 13% (*n* = 13/97) would be “very comfortable” with prescribing TNFi to women who may become pregnant within the next few years, respectively. Levels of comfort in prescribing TNFi were low among dermatologists regarding women actively planning pregnancy, pregnant women, and breastfeeding women.

**Fig. 8.**
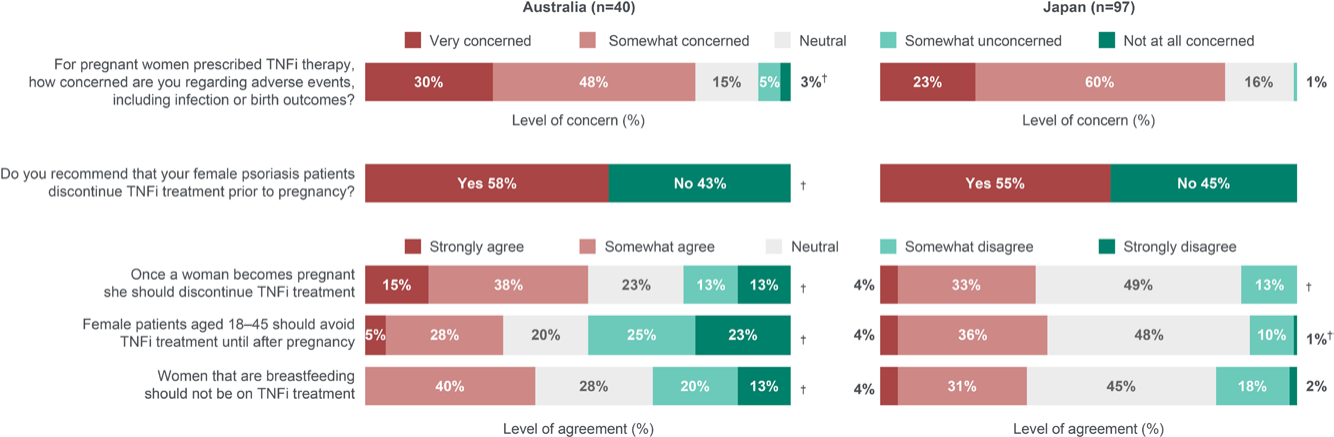
Dermatologists’ attitudes regarding concern over AEs and use of TNFi prior to pregnancy, during pregnancy, and during breastfeeding in women with PSO. Dermatologists from Japan were asked to disregard patients with psoriatic arthritis when responding. ^†^These data do not add up to 100% due to a rounding error. AEs, adverse events; PSO, psoriasis; TNFi, tumor necrosis factor inhibitors.

Approximately 80% of dermatologists overall expressed concern about adverse events, including infection and birth outcomes, among women who were prescribed TNFi during pregnancy (Fig. 8). Approximately one-third believed female patients aged 18 to 45 years in general should avoid TNFi until after pregnancy. Regarding women who were planning to become pregnant, more than half of dermatologists would recommend discontinuing TNFi prior to pregnancy. Among dermatologists from Australia and Japan, 53% (*n* = 21/40) and 37% (*n* = 36/97) agreed TNFi should be discontinued once a woman becomes pregnant, 40% (*n* = 16/40) and 35% (*n* = 34/97) agreed TNFi should be discontinued in women who are breastfeeding, and 68% (*n* = 27/40) and 47% (*n* = 46/97) discontinued TNFi in the majority of their patients that became pregnant (data not shown), respectively.

## Discussion

The patient survey demonstrated that most women felt they did not receive sufficient information from their HCPs to make informed decisions about disease management and pregnancy planning. Concurrently, most dermatologists were either impartial toward or not at all comfortable with prescribing TNFi to women aged 18 to 45 years. These findings collectively suggest a gap in information regarding the management of PSO and family planning in this group of patients.

### Providing greater support for family planning

Patients felt they lacked sufficient information and had to make a choice between treatment and family planning. Furthermore, most patients described that discussions around pregnancy planning were initiated by themselves or their partner, as opposed to being initiated by HCPs. A previous survey across the United States, Europe, and Japan similarly found that >75% of female patients aged 18 to 45 years with PSO initiated discussions on family planning with their HCPs.^18^ These results collectively suggest patients with PSO look to their dermatologists and other HCPs to provide information prior to pregnancy. Therefore, it is important to ensure dermatologists have an accurate understanding of TNFi in pregnancy and lactation so they can feel confident to proactively discuss family planning with their patients.

### Addressing concerns regarding TNFi use during pregnancy and lactation

In this survey, the patients’ treating physician was noted to be one of the main drivers of TNFi cessation during pregnancy. A proportion of women also reported feeling the need to choose between treatment and breastfeeding. However, at least half of all dermatologists surveyed were “not at all comfortable” with prescribing TNFi to women who were planning a pregnancy, pregnant, or breastfeeding.

These results may mirror local guidelines at the time the survey was conducted (2018–2020); for example, Japanese Dermatological Association 2019 guidance for the use of biologics in PSO recommends avoiding treatment among pregnant and breastfeeding women, though it acknowledges there are few reports to date suggesting toxicity or teratogenicity.^11^ In 2018, the Australasian Psoriasis Collaboration also recommended that, in the treatment of PSO in women who are planning a family, pregnant, or breastfeeding, drugs that have been used extensively are preferable to newer alternatives with less fetal safety data.^12^ In contrast, a 2023 update from the Australian Rheumatology Association indicated that, although TNFi are ideally withheld in the third trimester (with the exception of certolizumab pegol, which can be used throughout pregnancy), treatment may continue if clinically indicated.^19^ The update additionally noted women should be supported if they wish to breastfeed on TNFi.^19^ Similarly, 2019 guidelines by the American Academy of Dermatology and the National Psoriasis Foundation stated that TNFi are safe in pregnancy and during lactation.^10^

As more pregnancy, lactation, and pediatric-related TNFi safety data were cited as factors that would increase dermatologists’ comfort with prescribing TNFi among WoCBA, aligning international and local treatment guidelines could help allay concerns and fulfill gaps in knowledge about the safety of TNFi during pregnancy and lactation. Education for dermatologists can enable them to empower their patients in weighing the risk–benefit profile of compatible treatment options. This concurrently highlights a wider need for clear, accurate sources of biologic safety data on pregnancy and lactation for clinicians caring for WoCBA. To standardize knowledge and recommendations by dermatologists, further research into pregnancy and lactation safety of biologics (including TNFi) can build upon existing safety databases to ensure these resources are up-to-date and consistent. Institutions (eg, healthcare facilities) can collaborate with key stakeholders in the industry to drive research and establish further biologic safety data.

### Strengths

A key strength of the study relates to the insight it provides into both WoCBA with PSO and the current knowledge, attitudes, and practices of their dermatologists in the management of PSO. It is important to elucidate the concerns of dermatologists and recognize knowledge gaps or misconceptions, as these perceptions may ultimately influence the standard of care patients receive.

### Limitations

As the overall number of patients and dermatologists was small and response rates were not collected, results may not accurately reflect the views of all WoCBA with PSO or dermatologists. In terms of study design, there was a lack of formal validation for the questionnaires, which may affect the accuracy of the survey results. The patient and dermatologist surveys were also conducted 2 years apart, which could affect the validity of conclusions drawn from comparing perceptions between these groups, due to changing guidance on treatment options during this period. For example, further data on placental transfer of TNFi (eg, certolizumab pegol) were published shortly prior to the patient survey (conducted in 2018).^14,20^ As dermatologists were surveyed in 2020, the timing of these published data could have contributed toward differences in patient and dermatologist survey results on TNFi use during pregnancy. There was also a possibility of reporting or recall bias among participants (eg, self-reported disease severity and no verification of PSO diagnosis). Additionally, some of the survey questions were multiple response, allowing a single respondent to select more than one option. Therefore, the total number of responses received for some multiple-response questions may not exactly reflect the number of people who responded to the question, though this cannot be confirmed. Survey results were also limited to the attitudes and perceptions around TNFi and did not encompass other classes of treatment for PSO (eg, IL-23/IL-17 inhibitors, or other systemic medications), which could help provide a more complete picture of PSO management among WoCBA.

For the patient survey, women with unsuccessful pregnancies were excluded from the study, as the study sought to understand the participant’s perceptions throughout their pregnancy journey. However, it may be important to recognize that the experiences and perceptions of women with unsuccessful pregnancies may differ from those who had successful pregnancies, therefore affecting the generalizability of results to all WoCBA with PSO. Additionally, results would not capture perceptions from women planning to become pregnant but are not pregnant yet. Survey responses may also be skewed by the proportions of patients diagnosed with PSO before versus during pregnancy.

For dermatologists, demographic information was not consistently collected across both countries (eg, practice setting, level of familiarity with using TNFi in PSO, the proportion of PSO patients prescribed TNFi). Incorporation of such information would have been helpful to contextualize current TNFi prescribing behaviors and elucidate reasons for prescribing hesitancy. Additionally, only hospital-based dermatologists from Japan were included. Given PSO patients may subsequently be managed in a community setting, collecting responses from both hospital-based and community-based dermatologists could provide greater insight into the overall prescribing behaviors of the profession.

Finally, as data were only reported for Australia and Japan, differences in responses could be influenced by the social norms and cultures of each country. However, sociocultural influences cannot be reliably measured or compared, due to limitations of the survey design.

## Conclusion

Concerns and gaps in knowledge around family planning were common among WoCBA with PSO. Additionally, many dermatologists indicated that more TNFi safety data on pregnancy and lactation would increase their prescribing comfort in this group of patients.

To elicit changes in care commensurate with the needs of WoCBA with PSO, underlying dermatologist concerns regarding disease management among these patients need to be addressed. Educational resources improving dermatologists’ access to up-to-date, accurate biologic safety data (including that of TNFi) can guide risk versus benefit assessments for PSO management during patient consultations. This would enable dermatologists to support their patients in making informed decisions balancing effective disease management without compromise to family planning, and to discuss strategies that proactively safeguard the health of both mother and child.

## Conflicts of interest

The authors made the following disclosures: Y.Y.: Consulting fees, speaking fees, and grants from AbbVie, Boehringer Ingelheim, Bristol Myers Squibb, Eli Lilly Japan, Janssen, Kyowa Kirin, LEO Pharma, Maruho, Novartis, Taiho, and UCB. L.S.: Consultant, and/or scientific adviser, and/or investigator, and/or speaker for AbbVie, Amgen, Anacor, Ascend, Astellas, AstraZeneca, Blaze Bioscience, Boehringer Ingelheim, Botanix, Bristol Myers Squibb, Celgene, Dermira, Eli Lilly and Company, Galderma, Genentech, GSK, Hexima, Janssen, Leo Pharma, Mayne, Medimmune, Merck (MSD), Merck-Serono, Novartis, Otsuka, Pfizer, Phosphagenics, Photon MD, Regeneron, Roche, Samumed, Sanofi/Genzyme, SHR, Sun Pharma ANZ, Trius, UCB, and Zai Lab. Y.M.: Speaking fees from Abbvie, Eli Lilly Japan, Maruho, Sun Pharma Japan, and UCB. B.L. and A.L.: Employees of UCB and participants in the UCB Stock Award Plan. A.S.: Advisor, and/or speaking fees, and/or institutional support, and/or investigator in clinical trials for AbbVie, Amgen, Bristol Myers Squibb, Eli Lilly and Company, Janssen Cilag, Leo Pharma, Novartis, Pfizer, Sunpharma, and UCB.

## Funding

Supported by UCB. These surveys were conducted by market research companies, Hummingbird Insight and IQVIA, on behalf of UCB. Support for third-party writing assistance for this article, provided by Paige Foo Jia-Qi, MPharm, Costello Medical, Singapore, was funded by UCB in accordance with Good Publication Practice (GPP 2022) guidelines (https://www.ismpp.org/gpp-2022).

## Study approval

N/A

## Author contributions

YY, LS, YM, BL, AL, AS: Substantially contributed to study conception and design, analysis and interpretation of the data, drafting the article or revising it critically for important intellectual content, and final approval of the version of the article to be published.

## Data availability

The datasets generated during and/or analyzed during the current study are not publicly available as nonclinical studies are outside of the company’s data-sharing policy.

## Acknowledgements

The authors thank the patients and clinicians who took part in this study. The authors also acknowledge Irina Mountian, MD, PhD, UCB for publication coordination and Paige Foo Jia-Qi, MPharm, Costello Medical, Singapore, for medical writing and editorial assistance based on the authors’ input and direction.

## Presentation

This article includes data that have been presented as a poster and oral presentation at the 121^st^ Annual Meeting of the Japanese Dermatological Association (JDA), June 2–5, 2022, Kyoto, Japan.

## Supplementary data

Supplementary material associated with this article can be found at http://links.lww.com/IJWD/A62 and http://links.lww.com/IJWD/A61.

## Supplementary Material


