# Supplementary material for: Management of psoriasis in women 18 to 45 years of age in Australia and Japan: insights from patient and dermatologist surveys
Source: Int J Womens Dermatol. 2025 Jan 2;11(1):e189. doi: 10.1097/JW9.0000000000000189 (PMC11688090; doi:10.1097/JW9.0000000000000189)
Supplement: Supplementary file 1 [file jw9-11-e189-s001.pdf]

Management of Psoriasis in Women 18–45 Years of Age in Australia and Japan: Insights from Patient and Dermatologist Surveys

Supplementary Material

Supplementary Methods

Patient survey

Patients were recruited from consumer panels by Hummingbird Insight (Sydney, Australia) to participate in a 20-minute online survey (37 questions in total, including screening questions), conducted from September–October 2018. The surveys were developed in English and delivered in the local language of each country. Translation of the questionnaire for Japanese patients was also conducted by Hummingbird Insight (Sydney, Australia).

The patient questionnaire was adapted from a previous global survey, reported by Tincani et al. 2018.[1] This global survey was designed to identify how women balanced family planning and treatment of their condition, including topics such as quality of information received from healthcare professionals (HCPs), pre-conception planning, and treatment decisions around pregnancy and breastfeeding. The full patient questionnaire was reported by Tanaka et al. 2023, and adapted for this study with only slight differences in wording to account for the different patient groups.[2]

Dermatologist survey

A panel of dermatologists were contacted and invited by IQVIA (Sydney, Australia and Tokyo, Japan, for the respective countries) to complete an online survey (11 questions in total, including screening questions), conducted from October–December 2020. The surveys were developed in English and delivered in the local language of each country. Translation of the questionnaire for Japanese dermatologists was conducted by IQVIA (Tokyo, Japan).

The dermatologist questionnaire was also adapted from a previous global survey reported by Tincani et al. 2018.[3] This global survey was designed to assess dermatologists' comfort levels with prescribing TNFi for women aged 18–45 years, decisions around the use of a TNFi prior to, during, and after pregnancy, as well as concerns around adverse events associated with TNFi use during pregnancy. The full dermatologist questionnaire was reported by Tanaka et al. 2023, and adapted for this study with only slight differences in wording to account for the different HCPs.[2]

References

1. Tincani A, Taylor P, Fischer-Betz R, et al. FRI0693 Fears and misconceptions of women with chronic rheumatic diseases on their journey to motherhood. Ann Rheum Dis 2018;77:866.
2. Tanaka Y, Barrett C, Hirano Y, et al. Management of chronic rheumatic diseases in women 18–45 years of age in Asia Pacific: insights from patient and clinician surveys. Rheumatol Int 2023;43:721–33.
3. Tincani A, Taylor P, Fischer-Betz R, et al. FRI0692 Anti-TNF treatments for women with chronic inflammatory diseases: comparing attitudes and perceptions of physicians in Europe and the US. Ann Rheum Dis 2018;77:865.

1 *Patient questionnaire*

<table>
<tr><th>Question[a]</th><th>Response</th></tr>
<tr><td>1. When were you diagnosed with your (insert diagnosis)? [single response]</td><td>• Before your pregnancy
• During your pregnancy
• After your pregnancy</td></tr>
<tr><td>2. Did you ever use any medications for your (insert diagnosis)? [single response]</td><td>• Yes
• No</td></tr>
<tr><td>3. With which healthcare professional did you discuss at the different stages of your pregnancy? [multiple response]</td><td>
<table>
<tr><th></th><th>Before (when you start planning) your pregnancy</th><th>At the start of your pregnancy</th><th>During your pregnancy</th><th>After your pregnancy</th><th>In none of these situations</th></tr>
<tr><td>Dermatologists</td><td></td><td></td><td></td><td></td><td></td></tr>
<tr><td>GP</td><td></td><td></td><td></td><td></td><td></td></tr>
<tr><td>OBGYN</td><td></td><td></td><td></td><td></td><td></td></tr>
<tr><td>Paediatrician</td><td></td><td></td><td></td><td></td><td></td></tr>
<tr><td>Midwife</td><td></td><td></td><td></td><td></td><td></td></tr>
</table>
</td></tr>
<tr><td>3a. Before pregnancy, which one did you consider your most critical advisor? [single response, depending on answer in 3]</td><td>• Dermatologists
• GP
• OBGYN
• Paediatrician
• Midwife</td></tr>
<tr><td>4. Prior to pregnancy, during your consultation with this healthcare professional, who brought up the subject of family planning? [single response, depending on answer in 3a]</td><td>• Myself
• Physician/Healthcare professional from question 3a above
• My partner
• I do not remember</td></tr>
<tr><td>5. Can you still remember when the topic of planning a pregnancy was discussed for the first time with a healthcare professional? [single response]</td><td>• At the time of diagnosis
• At the time of treatment initiation
• At a regular visit
• I do not remember</td></tr>
<tr><td>6. If you think back about this discussion, to what extent did you … [select level]</td><td>
<table>
<tr><th></th><th>1 not at all</th><th>2</th><th>3</th><th>4</th><th>5</th><th>6</th><th>7</th><th>8</th><th>9</th><th>10 com-pletely</th></tr>
<tr><td>… feel like your questions and</td><td></td><td></td><td></td><td></td><td></td><td></td><td></td><td></td><td></td><td></td></tr>
</table>
</td></tr>
</table>

<table>
<tr><td></td><td><table>
<tr><td>concerns about pregnancy were addressed</td><td></td><td></td><td></td><td></td><td></td><td></td><td></td><td></td><td></td><td></td></tr>
<tr><td>…have the support of your healthcare professional on getting pregnant</td><td></td><td></td><td></td><td></td><td></td><td></td><td></td><td></td><td></td><td></td></tr>
</table></td></tr>
<tr><td>7. Did you have any concern that delayed your decision to become a mother for your most recent pregnancy? [single response]</td><td>• Yes
• No</td></tr>
<tr><td>8. Which (if any) of the following concerns delayed your decision to become a mother for your most recent pregnancy? [multiple response]</td><td>• I was not emotionally ready to become a parent
• My partner was not emotionally ready to become a parent
• I was not financially able to support a child/go on maternity leave
• My relationship with my partner was not in the right place to have a child
• I was not physically healthy enough to conceive and carry a child to term
• I might pass on a health issue to my child
• My education/career was not in the right place to have a child
• I thought I was too young to have a child
• I thought I was too old to have a child
• I did not have the support of my physician to become pregnant
• I did not have access to the medical care needed to become pregnant and give birth
• Other, please specify:
• None of the above</td></tr>
<tr><td>9. Which of the following best describes the circumstances around your most recent pregnancy?
[single response]</td><td>• I was actively trying to get pregnant
• I was open to seeing what happened (wasn't trying particularly hard one way or the other)
• I was not thinking about this
• I was actively trying to NOT get pregnant</td></tr>
<tr><td>10. How much time did it take you to become pregnant from the moment you started trying to conceive?</td><td>• ____ months
• I don't know</td></tr>
<tr><td>11. At the time you discovered you were pregnant, to what extent did you experience any of the following concerns? [single response]</td><td><table>
<tr><td></td><td>Not at all concerning</td><td></td><td></td><td></td><td>Very concerning</td><td>Not applicable</td></tr>
<tr><td>That the pregnancy might end in miscarriage</td><td></td><td></td><td></td><td></td><td></td><td></td></tr>
</table></td></tr>
</table>

That I was not physically healthy enough to be pregnant						
That my disease activity would compromise the health of the baby						
That I might pass on a health issue to my child						
That there might be something wrong with the child						
That pregnancy might make my disease worse						
That I did not have access to the medical care needed to have a successful pregnancy and give birth						
That the treatment I was on might harm the baby						

12. Prior to pregnancy, did you receive all the information you needed from your healthcare professional? Or was there any type of information you would have liked to receive and that you did not get? [multiple response]	• I received all the information I needed (fixed position)<br>• Impact of the disease activity on my health<br>• Impact of my disease activity on my baby<br>• Impact of the treatment on my baby<br>• Impact of pregnancy on my disease<br>• Other, please specify:
13. What treatment were you taking before your pregnancy? [multiple response]	• Immunosuppressants (e.g., ciclosporine)<br>• Topical products (e.g., calciprotriol, calcitriol, clobetasol, calcipetril/betamethasone)<br>• Tumour necrosis factor inhibitor (e.g., Enbrel [etanercept], Humira [adalimumab], Remicade [infliximab])<br>• Other biologic (including Stelara [ustekinumab], Cosentyx [secukinumab], Taltz [ixekizumab])<br>• Other oral (e.g., Otezla [apremilast])<br>• I don’t know
14. Before trying to conceive, did you and your treating physician create a treatment plan around what medications you would take and not take if you became pregnant? [single response]	• Yes<br>• No

<table>
<tr><td>15. Was there any discussion around your treatment plan between your treating physician and your obstetrician/gynaecologist? [single response]</td><td>Yes
No</td></tr>
<tr><td>16. Did you stop any of the following treatment and when? [single response]</td><td><table>
<tr><th></th><th>Before (when you start planning) your pregnancy</th><th>At the start of your pregnancy</th><th>During your pregnancy</th><th>After your pregnancy</th><th>In none of these situations</th></tr>
<tr><td>Same list as question 13</td><td></td><td></td><td></td><td></td><td></td></tr>
<tr><td></td><td></td><td></td><td></td><td></td><td></td></tr>
<tr><td></td><td></td><td></td><td></td><td></td><td></td></tr>
</table></td></tr>
<tr><td>17. You indicated you stopped your treatment. Whose idea was it to stop your treatment? [multiple response, based on Q3 and Q16 response]</td><td>My idea
My treating physician idea
My obstetrician/gynaecologist idea
My partner’s idea
Other, please specify:</td></tr>
<tr><td>18. What were the key reasons why your treatment was stopped during your most recent pregnancy?
[multiple response, based on Q16]</td><td>I/my physician [based on answer in Q17] was worried about the treatment harming the fetus
I/my physician [based on answer in Q17] could not find information if my treatment was compatible with pregnancy
My disease went into remission, so there was no need to treat
I/my physician [based on answer in Q17] believed disease would likely go into remission, so stopped treatment and waited to see if needed
I switched to a safer treatment/my physician switched me to a safer treatment [based on answer in Q17]
I/my physician [based on answer in Q17] worried about infection risk during labour
There was a possible contraindication with other medications
Physicians could not agree on the best plan
Other</td></tr>
<tr><td>19. How would you rate the severity of your (insert diagnosis) during your pregnancy? [single response]</td><td>Very well controlled/in remission
Well controlled/in low disease activity
Not very well controlled/in medium disease activity
Uncontrolled/in high disease activity</td></tr>
<tr><td>20. Did your disease improve, worsen or stay the same during your pregnancy? [single response]</td><td>Improved a lot
Improved a little
Stayed the same
Worsened a little</td></tr>
</table>

	• Worsened a lot
21. After your baby was born, did your disease improve, worsen or stay the same? [single response]	• Improved a lot<br>• Improved a little<br>• Stayed the same<br>• Worsened a little<br>• Worsened a lot
22. How many months after your baby was born did it worsen?	• ____ months after I gave birth
23. You indicated that you stopped your treatment before or during pregnancy. Did you reinitiate after your pregnancy? If so, how effective was the treatment when reinitiated? [single response, based on Q16]	• I did not reinitiate<br>• I did reinitiate and it was more effective<br>• I did reinitiate and it was less effective<br>• I did reinitiate and it was as effective as before<br>• I don't remember
24. Did you breast-feed your baby? [single response]	• Yes<br>• No
25. You indicated that you did not breast-feed your baby. Can you indicate what the reason you had for not doing so? [single response, based on Q24]	• Needed to start treatment for my illness immediately and could not breastfeed while on treatment<br>• Needed to go back to work<br>• Did not want to breastfeed<br>• Was physically unable to breastfeed<br>• Baby did not latch<br>• Doctor/healthcare professional recommended not to breastfeed<br>• Other, please specify:<br>• None of the above
26. Did your treating physician talk to you about the possibilities of breastfeeding? [single response]	• Yes, we talked about this for the first time before my pregnancy<br>• Yes, we talked about this for the first time during my pregnancy<br>• Yes, we talked about this for the first time right after delivery<br>• No, this option was not discussed
27. Which other healthcare professional(s) discussed the possibilities of breastfeeding with you? [multiple response]	• Primary Care Physician/General Practitioner<br>• Obstetrician or Gynaecologist<br>• Paediatrician<br>• Lactation Specialist<br>• Midwife<br>• Other, please specify:<br>• I didn't consult a healthcare professional at this point
28. Can you also indicate which of the healthcare professionals was the most important in	• Primary Care Physician/General Practitioner<br>• Dermatologist<br>• Obstetrician or Gynaecologist

influencing your decision whether or not to breastfeed?
[single response, based on Q26 and Q27 response]

- Paediatrician
- Lactation Specialist
- Midwife
- Other, please specify:

29. To what extent did you and/or your healthcare professionals worry about the consequences of your treatment on your baby while breastfeeding? [multiple response, based on Q23 response]

	1 not at all concerned									10 very concerned
Myself										
My gynaecologist or paediatrician or lactation specialist										
Dermatologist										

30. Did you feel you had to make a choice between breastfeeding and treatment? [multiple response]

- Yes, I had the feeling that I could not combine both breastfeeding and my treatment
- No, I had the feeling that I could combine both breastfeeding and my treatment

31. How reliable do you feel the information from your healthcare professional(s) is on how to manage your disease during pregnancy? [single response per row]

	1 Not at all reliable	2	3 Neither reliable, nor unreliable	4	5 Very reliable	Not applicable for me
Primary Care Physician/General Practitioner						
Midwife						
Dermatologist						
Obstetrician or Gynaecologist						
Paediatrician						
Other; please specify […]						

32. How consistent do you feel the information you received from different healthcare

	1 Very conflicting	2	3 Neither conflicting,	4	5 Very consistent	I did not receive any

professionals is regarding the following? [single response per row]				nor consistent			information on this topic
	Medications to take during pregnancy						
	What to do prior to becoming pregnant						
	How to manage pregnancy overall						
	When to stop medications						
	Risk of medication on your pregnancy outcome						
	Medications to take during breastfeeding						

Adapted from Tanaka et al. Rheumatol Int 2023;43:721–33. [a]Screening and demographic questions are not included.

1 *Dermatologist questionnaire*

<table>
<tr><th>Question[a]</th><th>Response</th></tr>
<tr><td>Q1. What percentage of your female patients with psoriasis fall into the following age groups? [numeric response; sum up to 100%]</td><td>• <18 years old
• 18–35
• 36–45
• 46–64
• 65+</td></tr>
<tr><td>Q2. What percentage of your female patients between the age of 18–45 with psoriasis do you currently prescribe a TNFi treatment? [numeric response; range 0–100%]</td><td>• Please enter a number</td></tr>
<tr><td>Q3. How comfortable are you in prescribing TNFi therapy for the following types of patients? [single response per row]</td><td>
<table>
<tr><th></th><th>Not at all comfortable</th><th>Neutral</th><th>Very comfortable</th></tr>
<tr><td>All female patients between the age of 18–45</td><td>1</td><td>2</td><td>3</td></tr>
<tr><td>Female patients who may become pregnant within the next few years</td><td>1</td><td>2</td><td>3</td></tr>
<tr><td>Female patients who are actively trying to become pregnant</td><td>1</td><td>2</td><td>3</td></tr>
<tr><td>Female patients who are pregnant</td><td>1</td><td>2</td><td>3</td></tr>
<tr><td>Female patients who are breast feeding</td><td>1</td><td>2</td><td>3</td></tr>
</table>
</td></tr>
<tr><td>Q4. Do you recommend that your female patients discontinue a TNFi agent prior to pregnancy? [single response]</td><td>• Yes
• No</td></tr>
<tr><td>Q5. How many months prior to pregnancy would you recommend that TNFi therapy is discontinued? [single response, based on Q4 response]</td><td>• Immediately prior / as soon as pregnancy is suspected and/or confirmed
• Up to 3 months prior
• 3 to less than 6 months prior
• 6 to less than 9 months prior
• 9 to less than 12 months prior
• 12 months+ prior</td></tr>
</table>

<table>
<tr><td>Q6. For your female patients who become pregnant while they are taking a TNFi agent, in what percentage of patients is the decision made to discontinue her biologic treatment? [single response]</td><td>For less than 10% of patients
For 10% to less than 25% of patients
For 25% to less than 50% of patients
For 50% to less than 75% of patients
For 75% or more of patients</td></tr>
<tr><td>Q7. Hypothetically, what, if anything, would make you more comfortable with using TNFi agents among female patients between the age of 18-45 who may become pregnant in the future? [multiple response]</td><td>More safety data for child 5 years post delivery
More safety data during pregnancy
More lactation data
A multidisciplinary discussion (Obstetrician etc).
Recommendation from a Scientific Leader (Key Opinion Leader)
Nothing</td></tr>
<tr><td>Q8. How strongly do you agree with the following statements? [single response per row]</td><td><table>
<tr><th></th><th>Strongly Disagree</th><th>Somewhat Disagree</th><th>Neutral</th><th>Somewhat Agree</th><th>Strongly Agree</th></tr>
<tr><td>Female patients 18-45 should avoid TNFi therapies until after pregnancy</td><td>1</td><td>2</td><td>3</td><td>4</td><td>5</td></tr>
<tr><td>Once a woman becomes pregnant she should discontinue TNFi treatment</td><td>1</td><td>2</td><td>3</td><td>4</td><td>5</td></tr>
<tr><td>Women that are breastfeeding should not be on a TNFi agent</td><td>1</td><td>2</td><td>3</td><td>4</td><td>5</td></tr>
<tr><td>If a patient’s disease is controlled during pregnancy, it reduces the risk of pregnancy complications</td><td>1</td><td>2</td><td>3</td><td>4</td><td>5</td></tr>
<tr><td>Keeping auto-immune disease controlled during pregnancy is my primary goal</td><td>1</td><td>2</td><td>3</td><td>4</td><td>5</td></tr>
<tr><td>During pregnancy I typically co-manage the auto-immune disease with other specialities (e.g. Obstetricians and Gynaecologists, Primary Care Physicians etc.)</td><td>1</td><td>2</td><td>3</td><td>4</td><td>5</td></tr>
<tr><td>If there was a TNFi that could be used throughout pregnancy, I would be comfortable that my patients stay under treatment with this TNFi throughout their pregnancy</td><td>1</td><td>2</td><td>3</td><td>4</td><td>5</td></tr>
</table></td></tr>
<tr><td>Q9. For women who are pregnant and are prescribed TNFi therapy, how concerned are you</td><td>Very concerned
Somewhat concerned
Neutral</td></tr>
</table>

regarding adverse events, including infection or birth outcomes? [single response]	• Somewhat unconcerned<br>• Not at all concerned

Adapted from Tanaka et al. Rheumatol Int 2023;43:721–33. [a]Screening and demographic questions are not included.

Supplementary Tables

Table S1. Patient demographics and disease characteristics

Patients	Australia (n=27)	Japan (n=31)
**Age group, years, n (%)**		
18–30	6 (22)	7 (23)
31–40	20 (74)	19 (61)
41–45	1 (4)	5 (16)
**Pregnant, n (%)**		
In the past 2 years	13 (48)	14 (45)
2–5 years ago	14 (52)	17 (55)
**Self-reported PSO severity, n (%)**		
Moderate	18 (67)	29 (94)
Severe	9 (33)	2 (6)
**Time of PSO diagnosis, n (%)**		
Before pregnancy	11 (41)	29 (94)
During pregnancy	16 (59)	2 (6)
**Medications taken before pregnancy, n (%)**		
TNFi[a]	15 (56)	6 (19)

[a] The survey question did not specify the indication as PSO ('What treatment were you taking before your pregnancy?'). PSO: psoriasis; TNFi: tumor necrosis factor inhibitors.

Table S2. Dermatologist demographics

Dermatologists	Australia (n=40)[a]	Japan (n=97)
**Age group, years, n (%)**		
<40	4 (10)	43 (44)
40–<50	10 (26)	32 (33)
≥50	19 (49)	22 (23)
Prefer not to answer	6 (15)	0
**Gender**		
Male	18 (46)	68 (70)
Female	20 (51)	29 (30)
Prefer not to answer	1 (3)	0

[a] All percentages for demographic data were calculated for n=39 respondents from Australia, as this information was not available for 1 respondent.

Supplementary Figures

Figure S1. Breastfeeding among women with PSO

a. **Proportion of women with PSO that did not breastfeed their baby**

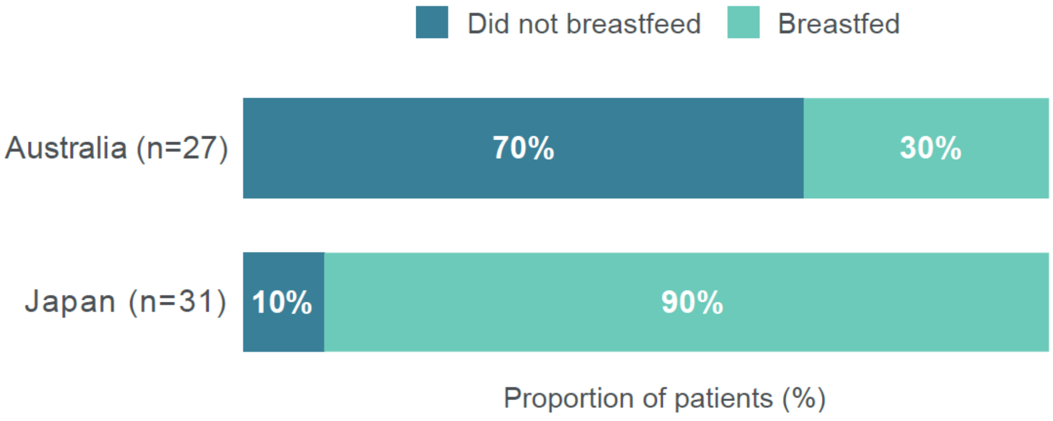

b. Patient-reported reasons for not breastfeeding

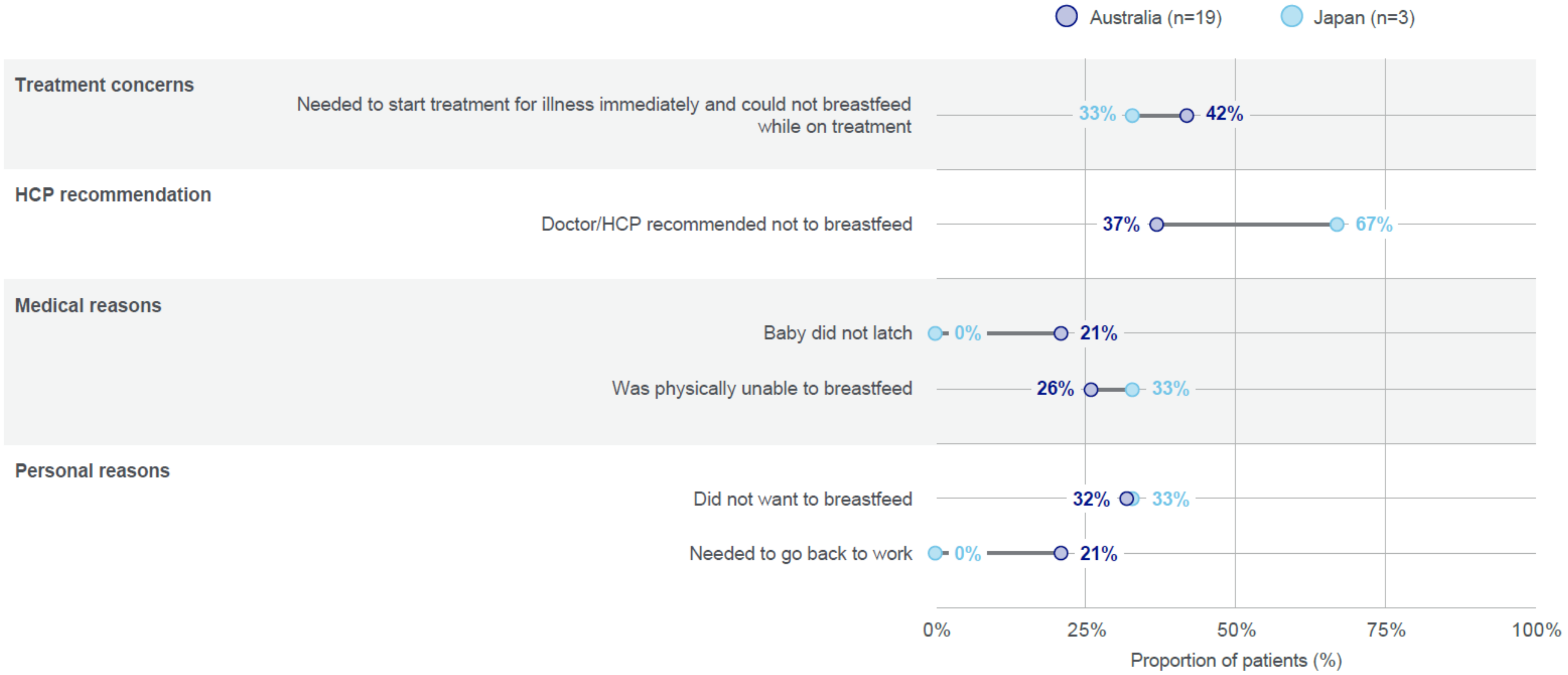

Data should be interpreted with caution due to small n numbers. (a) 'Did you breastfeed your baby?'; (b) 'You indicated that you did not breastfeed your baby. Can you indicate what reasons you had for not doing so?' Results reported for those who indicated 'no' in question (a). All possible answers are shown, except for the 'None of the above' (1 [5%] response from an Australian patient and 1 [33%] response from a Japanese patient) and 'Other' categories (no responses were recorded). HCP: healthcare professional; PSO: psoriasis.
