# Supplementary material for: Management of psoriasis in women 18 to 45 years of age in Australia and Japan: insights from patient and dermatologist surveys
Source: Int J Womens Dermatol. 2025 Jan 2;11(1):e189. doi: 10.1097/JW9.0000000000000189 (PMC11688090; doi:10.1097/JW9.0000000000000189)
Supplement: Supplementary file 2 [file jw9-11-e189-s002.pdf]

Management of Psoriasis in Women 18–45 Years of Age in Australia and Japan: Insights from Patient and Dermatologist Surveys

Yukie Yamaguchi, Lynda Spelman, Yoko Mizutani, Bartosz Lukowski, Alfred Lanzafame, Annika Smith

In Australia and Japan, there are limited data on the perspectives of women of childbearing age with psoriasis (PSO) and dermatologists, particularly on tumor necrosis factor inhibitor (TNFi) use

Identifying patient- and dermatologist-perceived barriers to treatment among women of childbearing age with PSO can guide disease management

Online surveys were completed by

Women aged 18–45 years
Pregnant within the past 5 years with self-reported moderate to severe PSO

Dermatologists
Managing PSO with biologics in at least one female patient aged 18–45 years

Concerns and gaps in information around family planning were common among women of childbearing age with PSO

Dermatologists had **safety concerns around prescribing TNFi in pregnancy**

- **Less than half** of women felt they received all the information they needed prior to pregnancy
- **One third** of women delayed their decision to have children, most commonly due to **hereditary concerns**

Safety-related concerns were among the most common reasons for stopping TNFi during pregnancy

More safety data on pregnancy, breastfeeding, and pediatric post-delivery outcomes would increase dermatologist comfort with prescribing TNFi

There is a need to address both patient and dermatologist concerns regarding family planning and disease management among women of childbearing age with PSO

Improving access to up-to-date, accurate TNFi safety data can enable dermatologists to support patients in making informed decisions balancing disease management without compromise to family planning, and to discuss strategies that proactively safeguard the health of both mother and child
